# Parvovirus B19 1A complete genome from a fatal case in Brazil

**DOI:** 10.1590/0074-02760150261

**Published:** 2015-09

**Authors:** Liliane Costa Conteville, Louise Zanella, Michel Abanto Marín, Ana Maria Bispo de Filippis, Rita Maria Ribeiro Nogueira, Ana Carolina Paulo Vicente, Marcos César Lima de Mendonça

**Affiliations:** 1 Fundação Oswaldo Cruz, Instituto Oswaldo Cruz, Laboratório de Genética Molecular de Microrganismos, Rio de Janeiro, RJ, Brasil; 2 Fundação Oswaldo Cruz, Instituto Oswaldo Cruz, Laboratório de Flavivírus, Rio de Janeiro, RJ, Brasil

**Keywords:** parvovirus B19, genotype 1 genome, fatal case

## Abstract

Parvovirus B19 (B19V) infects individuals worldwide and is associated with an ample range of pathologies and clinical manifestations. B19V is classified into three distinct genotypes, all identified in Brazil. Here, we report a complete sequence of a B19V genotype 1A that was obtained by high-throughput metagenomic sequencing. This genome provides information that will contribute to the studies on B19V epidemiology and evolution.

Primate erythroparvovirus 1, previously referred to as Parvovirus B19 (B19V), is a single-stranded linear DNA nonenveloped virus that belongs to the family Parvoviridae and genus Erythroparvovirus (Adams et al. 2014). B19V infects individuals worldwide and is the etiological agent associated with erythema infectiosum, aplastic crisis, hydrops faetalis and arthritis; in rare cases it has been associated to co-infections in human immunodeficiency virus-positive patients, acute leukaemias in children and generalised oedema in adults (Kerr et al. 2003, Pereira et al. 2014, Vlaara et al. 2014).

The genome of B19V is about 5.6 kb with two major open reading frames (ORFs) flanked by two inverted terminal repeats (ITRs), that can be folded into hairpins and are involved in virus replication (Cotmore & Tattersall 2005). One ORF encodes a nonstructural protein (NS1) and the other one, two capsid proteins (VP1 and VP2). VP1 and VP2 share the same amino acid (aa) sequence, but VP1 has an unique region (VP1u) at the amino terminus represented by an additional 227 aas. Besides these major ORFs, there are three minor ORFs that encode NSs: 7.5 kDa, X and 11 kDa. All transcripts are expressed from a single promoter, the p6 promoter (Ozawa et al. 1987, Zhi et al. 2006).

B19V was classified into three distinct genotypes (1, 2 and 3) based on NS1-VP1u region. Genotype 1 was segregated into subtypes 1a and 1b and genotype 3 into subtypes 3a and 3b (Servant et al. 2002, Toan et al. 2006, Parsyan et al. 2007). All three genotypes have been identified in Brazil (Sanabani et al. 2006) but, so far, there are only nearly full-length genome sequences of B19V, most of them are from patients in São Paulo, with different types of leukaemia (da Costa et al. 2013).

In this study we revealed the first full genome of a B19V genotype 1A from a fatal case of a 12-year-old boy from Rio de Janeiro, Brazil with suspected dengue infection. This genome was recovered from a serum sample by metagenomic approach using high-throughput sequencing performed in Illumina HiSeq 2500 platform. Taxonomic profiling programs found hits with similarity to B19V. *de novo* assembly was performed with SPAdes 3.5.0. Specific PCR and Sanger sequencing confirmed the presence of B19V in the sample. Phylogenetic analysis was performed using NS1-VP1-VP2 regions and showed that the B19V/RJ2929 strain belongs to genotype 1A (data not shown).

The B19V/RJ2929 genome is 5,594 bp in length with overall 43.92% GC content (Figure). Contrasting, the ITRs (inferred from published sequence FN598217) have higher GC content (57.85%), resulting in a stable hairpins formation used as a self-primer to start genome replication. All binding sites for transcriptional factors of the p6 promoter are conserved. The comparison of B19V/RJ2929 with B19V 1A sequences available in GenBank revealed some aa substitutions in the major and minor proteins: in NS1, F444C and M452I, two conservative substitutions, in VP1-VP2, two nonconservative substitutions P740R and T741P and in 11 kDa there was one conservative substitution D65N.

This complete genome has been deposited in GenBank under accession KT268312.

**Figure:**
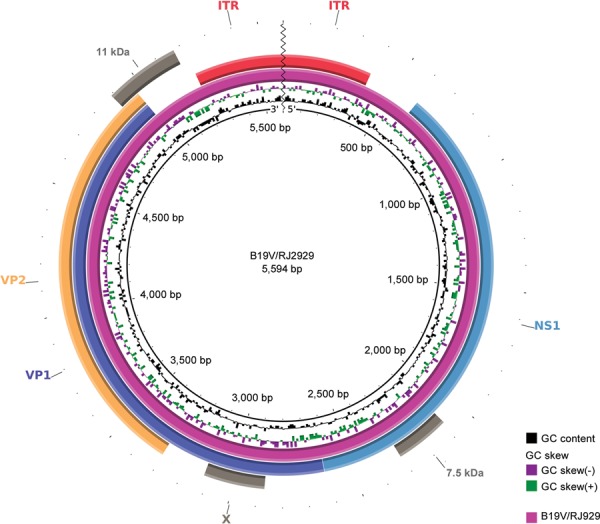
Genomic map of Parvovirus B19 (B19V). The inner circle represents 5’-3’ sequence sense followed by percentual GC content and GC skew. B19V/RJ2929 genome is the purple circle. Major and minor open reading frames and inverted terminal repeats (ITRs) are labelled. Figure was performed using Blast Ring Image Generator (sourceforge.net/projects/brig). NS: nonstructural protein.
